# Characterization of the seminal plasma proteome in men with prostatitis by mass spectrometry

**DOI:** 10.1186/1559-0275-9-2

**Published:** 2012-02-06

**Authors:** Daniel Kagedan, Irene Lecker, Ihor Batruch, Christopher Smith, Ilia Kaploun, Kirk Lo, Ethan Grober, Eleftherios P Diamandis, Keith A Jarvi

**Affiliations:** 1 Department of Pathology and Laboratory Medicine, Mount Sinai Hospital, Toronto, Canada; 2 Division of Urology, Department of Surgery, Mount Sinai Hospital, University of Toronto, Toronto, Canada; 3 Department of Pathology and Laboratory Medicine, Samuel Lunenfeld Research Institute, Mount Sinai Hospital, Toronto, Canada; 4 Department of Laboratory Medicine and Pathobiology, University of Toronto, Toronto, Canada; 5 Institute of Medical Sciences, University of Toronto, Toronto, Canada

**Keywords:** prostatitis, seminal plasma, inflammation, biomarkers, mass spectrometry

## Abstract

**Background:**

Prostatitis is an inflammation of the prostate gland which affects approximately 10% of men. Despite its frequency, diagnosing prostatitis and monitoring patient response to treatment remains frustrating. As the prostate contributes a substantial percentage of proteins to seminal plasma, we hypothesized that a protein biomarker of prostatitis might be found by comparing the seminal plasma proteome of patients with and without prostatitis.

**Results:**

Using mass spectrometry, we identified 1708 proteins in the pooled seminal plasma of 5 prostatitis patients. Comparing this list to a previously published list of seminal plasma proteins in the pooled seminal plasma of 5 healthy, fertile controls yielded 1464 proteins in common, 413 found only in the control group, and 254 found only in the prostatitis group. Applying a set of criteria to this dataset, we generated a high-confidence list of 59 candidate prostatitis biomarkers, 33 of which were significantly increased in prostatitis as compared to control, and 26 of which were decreased. The candidates were analyzed using Gene Ontology and Ingenuity Pathway analysis to delineate their subcellular localizations and functions.

**Conclusions:**

Thus, in this study, we identified 59 putative biomarkers in seminal plasma that need further validation for diagnosis and monitoring of prostatitis.

## Background

Prostatitis is a very common condition, with symptoms affecting approximately 10% of all men [1]. Asymptomatic prostatitis likely affects many more men. Prostatitis is the most common urologic diagnosis in males younger than 50 years, and the third most common diagnosis in males older than 50. Annually, prostatitis accounts for approximately 2 million outpatient visits to urology practices in the United States alone [2].

The symptoms associated with prostatitis are extremely variable in location, severity, duration and type. The symptom complex includes increased urinary frequency, urgency, and dysuria, as well as pain in the pelvic region, penis and urethra. The symptoms range from mild to disabling and may be of limited duration, or chronic, with episodic worsening. With such a variable phenotype, it is not surprising that prostatitis is a challenge to diagnose and treat.

Prostatitis may also be asymptomatic, with an adverse effect on fertility. This condition has been associated with male infertility through a mechanism involving inflammatory cytokines and their negative impact on fertility [3,4].

Currently, prostatitis is diagnosed using symptom questionnaires, digital rectal exam, and by culturing urine samples and expressed prostatitis secretions.

Despite the National Institutes of Health classification system [5], diagnosing prostatitis remains confusing and frustrating to urologists, as many of the symptoms overlap. Moreover, diagnostic tests fail to reliably differentiate one type of prostatitis from another, and even fail to distinguish prostatitis from healthy asymptomatic individuals [6]. There are no biomarkers to diagnose this condition accurately or to follow the course of therapy.

One potential method for diagnosing prostatitis involves testing bodily fluids for biomarkers of the disease. As prostatic secretions form a substantial proportion (25%) of the semen, seminal plasma is an excellent fluid to search for such markers of prostatic inflammation. The rest of the seminal plasma is produced by the seminal vesicles (65%), testes and epididymis (10%), and periurethral glands (minimal) [7].

Prostatic proteins can enter semen through several pathways including: 1) normal secretory pathways; 2) release of membrane-bound structures in seminal plasma called prostasomes; and 3) via epithelial shedding in which abraded epithelial cells shed their protein content into seminal plasma [8]. The latter pathway results in intracellular proteins entering the semen, and holds enormous potential in identifying biomarkers of diseases affecting specific tissues, by analyzing the proteins leaked from cells of those tissues. In fact, the most commonly used biomarker for prostate diseases, prostate-specific antigen (PSA), in wide use as a biomarker of prostate cancer, was originally found and isolated from the semen [9].

There have been previous attempts to identify biomarkers of prostatitis in the semen. Several studies have examined the presence of leukocytes or bacteria in semen of men with prostatitis [10-13]. However, the vast majority of prostatitis is abacterial (or at least non-cultureable), and increased leukocytes in the semen can be due to inflammation at other locations within the male urogenital tract [13]. Furthermore, leukocytospermia is unable to detect all cases of urogenital inflammation, and hence other markers are needed for an accurate diagnosis of inflammation specific to the prostate gland [14].

Other studies have investigated the potential of inflammatory proteins and cytokines as prostatitis biomarkers. Specifically, interleukin-8 [15-17], interleukin-6 [17], interleukin-10, immunoglobulin-A [18], elastase [12,19], peroxidase-positive leukocytes [19], and nerve growth factor [20] were found to be significantly altered in the seminal plasma of patients with prostatitis or chronic pelvic pain syndrome. However, none of these proteins is definitive in arriving at a diagnosis.

There have been no extensive studies on the protein composition of semen in men with prostatitis. The above studies measured levels of certain inflammatory proteins, making it possible that potential biomarkers which are not directly measured would have been missed.

There are very few studies on semen proteins: Pilch and Mann found 923 proteins in the semen of a healthy man [8]. We recently performed mass spectrometry on semen samples from men with infertility and healthy fertile controls and identified approximately 2300 unique proteins [21].

We hypothesized that seminal plasma from men with prostatitis may contain noticeably different concentrations of specific proteins that could be used as biomarkers to diagnose prostatitis and monitor its response to treatment. In addition to the above, a prostatitis biomarker would be invaluable in distinguishing prostatitis from other causes of increased serum PSA levels, decreasing unnecessary anxiety and invasive investigations for men with elevated PSA [22,23].

By using a shotgun proteomic approach, we compared the proteomes of seminal plasma from men with confirmed prostatitis to the proteomes of seminal plasma from fertile, healthy controls [21]. Seminal plasma samples from 5 individuals from each of the two diagnostic groups were pooled together to account for inter-individual variation in protein composition. Using this approach, we compiled a list of candidate prostatitis biomarkers and verified two of them using an ELISA assay.

## Results and discussion

### LC-MS analysis

Following trypsin digestion, each replicate was fractionated using SCX liquid-chromatography to increase the number of peptides identified in each fraction. Three replicates were analyzed in order to gauge reproducibility, as well as to increase protein identification and prediction confidence. The first 10 fractions from SCX were analyzed using an 88 min gradient and the next 11 fractions were analyzed using a 55 min gradient, since the first 10 fractions had greater sample complexity, allowing for more peptide identifications (data not shown).

### Data analysis

Data was searched with Mascot and X!Tandem to increase sequence coverage and the number of proteins identified. Search results were merged using Scaffold 2.0 in MudPit mode for analysis and visualization. Mascot and X!Tandem filter settings were adjusted to achieve a FPR of 1.1-1.5%. A triplicate sample dataset was uploaded into Scaffold. The original Scaffold file (Prostatitis.sfd) can be downloaded from Tranche (http://www.ProteomeCommons.org) hash code: AAlPx7YK40+620DDQaBE2qOoMTkT7LqKHsEDFO/pc/WDtaIRrYEn9bIEAtQ+7q7WPGOGZhDmSebpgfJbUEdHg6pNUXQAAAAAAAACjQ==).

**Figure 1:**
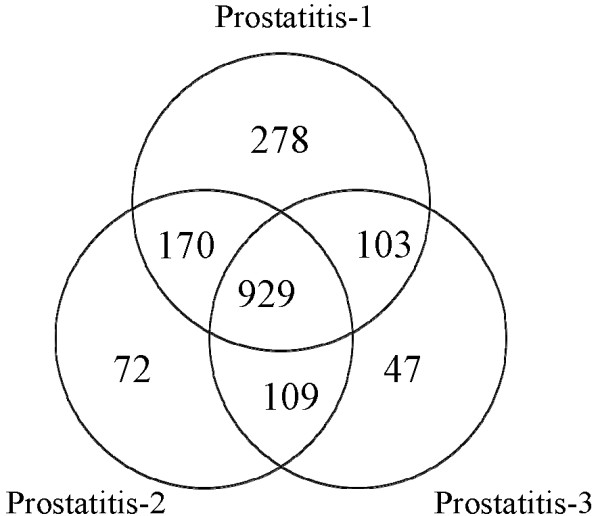
**Venn diagram representing overlap of identified proteins among triplicate seminal plasma samples in prostatitis patients**. 1708 proteins identified in total.

In triplicate analysis of the Prostatitis group we found a total of 1708 proteins (including 9 proteins that matched reverse sequences) at a FPR of 1.1% with 1449, 1357 and 1157 proteins in each replicate (Figure 1, Additional File 1).

### Comparison of Control and Prostatitis protein lists

**Figure 2:**
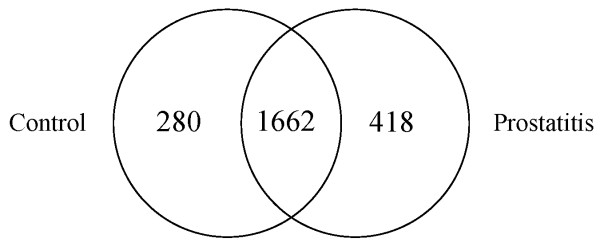
**Venn diagram representing overlap of proteins identified in prostatitis vs control (21) diagnostic groups**.

Comparing Control and Prostatitis proteomes, there are 1464 proteins in common, 413 found only in Control and 254 found only in Prostatitis (Figure 2, Additional File 2). In total, we identified 2131 proteins (including 12 proteins that matched reverse sequences) in the combined Control and Prostatitis groups with a FPR of 1.1%. The spectrum report for the Prostatitis dataset may be obtained by following the instructions outlined in Additional File 3.

Proteins that had their counts significantly affected due to shared peptides were carefully evaluated and removed from further analysis. Spectral counting is a semi-quantitative method with the advantage of having a linear dynamic range of several orders of magnitude [24]. Due to the inherent limitations of this quantitative method which include poor sensitivity to small changes in abundance (or sensitivity to fold changes greater than 100%) and diminished quantitative accuracy of proteins identified by very few counts [24,25], the following criteria were used to select a high-confidence list of potential proteins with significantly increased concentration in one of the groups: a) Fold differences must be greater than or equal to 2; b) The coefficient of variation (CV) for the 3 replicates must be less than or equal to 50% if the average spectral count value is > 5; c) If one clinical group has a spectral count average ≤ 5, then a CV of less than 100% for that group was accepted; d) If one clinical group has zero spectral counts, then the other clinical group must have an average spectral count > 5; e) If one clinical group has an average spectral count < 10, then the other must have an average spectral count > 10. By applying the above mentioned selection criteria, we found the following: 33 proteins were significantly increased in prostatitis as compared to controls (Table 1), and 26 proteins were significantly increased in controls as compared to prostatitis (Table 2).

**Table 1:** List of candidate biomarker proteins found at higher abundance in prostatitis compared to control, according to spectral counting.

Gene Name	Average Spectral Count		Fold Change*	Prostatic Origin**
	**Control**	**Prostatitis**		

ALB	1441	3079	2.1	

SERPINA1	149	382	2.6	Yes

CST4	83	468	5.7	

CST3	65	202	3.1	Yes

PAEP cDNA FLJ52183	52	144	2.8	

C4A	38	131	3.5	Yes

A2M	44	103	2.3	Yes

SERPINA5	37	122	3.3	

C3	34	104	3	Yes

LPL	30	66	2.2	

IGHG2	17	46	2.6	

A1BG	25	50	2	

MSLN	16	33	2	

SERPING1	17	48	2.9	

SCPEP1	17	44	2.6	Yes

GLA	21	43	2	Yes

OLFM4	15	41	2.7	Yes

SMPD1	9	20	2.2	Yes

CTSF	9	24	2.5	Yes

SERPINF1	10	33	3.4	Yes

MINPP1	9	19	2.1	Yes

CFI	9	18	2.1	

CTSC	5	10	2.2	

CST6	7	15	2.2	Yes

TXNDC16	8	18	2.3	Yes

LCN1	2	15	6.3	

COL6A2	3	15	5.5	Yes

A4GALT	4	15	4	Yes

PATE4	4	13	3	Yes

TIMP3	5	11	2.1	

CST2	2	16	9.6	

FGB	4	11	2.6	

COMP	4	12	2.9	

* Fold change = average prostatitis/average control

** Protein/gene name annotated as expressed in the prostate in at least 2 out of 3 databases (BioGPS; Unigene, Protein Atlas)

**Table 2:** List of candidate biomarker proteins found at higher abundance in control compared to prostatitis, according to spectral counting.

Gene Name	Average Spectral Count		Fold Change*	Prostatic Origin**
	**Control**	**Prostatitis**		

CPM	31	10	3	Yes

DYNC1H1	29	13	2.3	

MUC5B	29	7	4.2	

RAB27B	21	11	2	Yes

PHGDH	20	10	2.1	Yes

CA2	19	9	2	

ACO1	14	6	2.3	Yes

ANXA6	19	5	3.5	

CACNA2D1	19	5	4	

FAM129A	10	4	2.4	Yes

CDC42	12	4	2.7	Yes

RHOC	13	4	3.2	Yes

HGD	14	5	2.9	

RAB2A	11	5	2.1	Yes

DCXR	12	6	2.1	Yes

LAMP1	10	4	2.3	Yes

ALDH9A1	11	5	2	Yes

CMPK1	13	5	2.5	

NOV	13	5	2.4	Yes

NAPA	12	4	2.8	Yes

SOD3	12	5	2.3	Yes

LIFR	11	5	2.3	Yes

RNPEP	12	5	2.2	Yes

FLNB	13	4	3.2	Yes

DDAH1	12	5	2.3	Yes

KIF5B	11	4	3.1	Yes

* Fold change = average control/average prostatitis

** Protein/gene name annotated as expressed in the prostate in at least 2 out of 3 databases (BioGPS; Unigene, Protein Atlas)

To identify the tissue specificity of the candidate prostatitis biomarkers, candidates were searched against UniGene, BioGPS, and Human Protein Atlas. According to UniGene, 18 proteins had "strong" expression in the prostate, 19 had "moderate" expression in the prostate, and 12 had "weak" expression in the prostate. According to Human Protein Atlas, 10 proteins had "strong" expression in the prostate, 11 proteins had "moderate" expression in the prostate, 11 had "weak" expression in the prostate, and the remainder were not expressed in the prostate. According to BioGPS, which catalogues mRNA expression in various tissues, 19 proteins have strong gene expression in the prostate, as defined by an expression of 3 times the average tissue expression.

**Figure 3:**
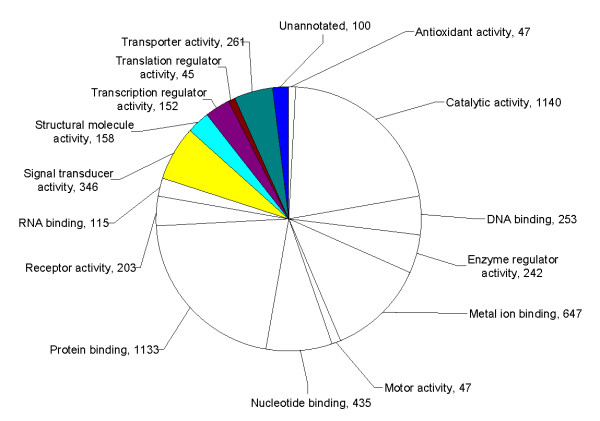
**Pie chart representing seminal plasma proteome of prostatitis patients in terms of molecular functions**.

**Figure 4:**
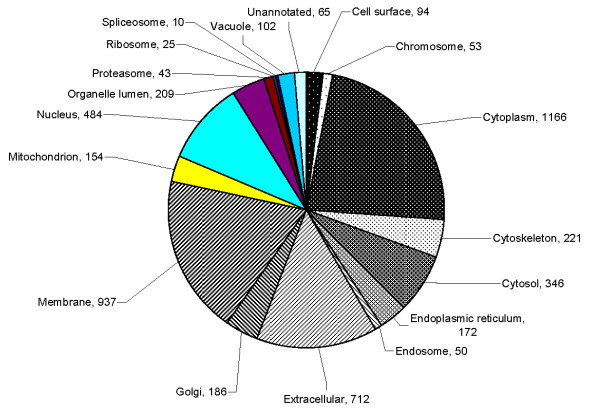
**Pie chart representing seminal plasma proteome of prostatitis patients in terms of cellular components**.

**Figure 5:**
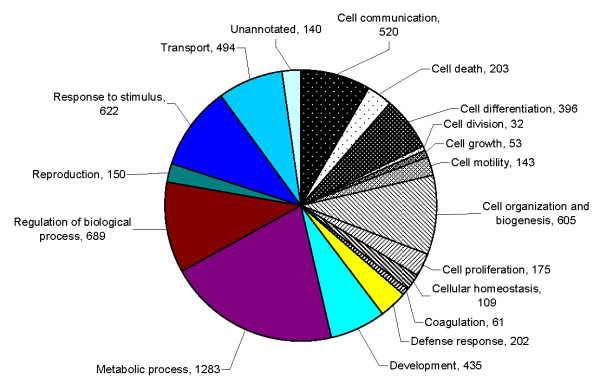
**Pie chart representing seminal plasma proteome of prostatitis patients in terms of biological processes**.

The seminal plasma proteome of prostatitis patients was analyzed using ProteinCenter software to ascertain the Gene Ontology (GO) annotations for molecular functions (Figure 3), cellular components (Figure 4), and biological processes (Figure 5) for the entire prostatitis proteome. The top 3 molecular functions were catalytic activity, protein binding, and metal ion binding. The top 3 cellular components were cytoplasm, membrane, and extracellular. The top 3 biological processes were metabolic processes, regulation of biological processes, and response to stimulus.

**Figure 6:**
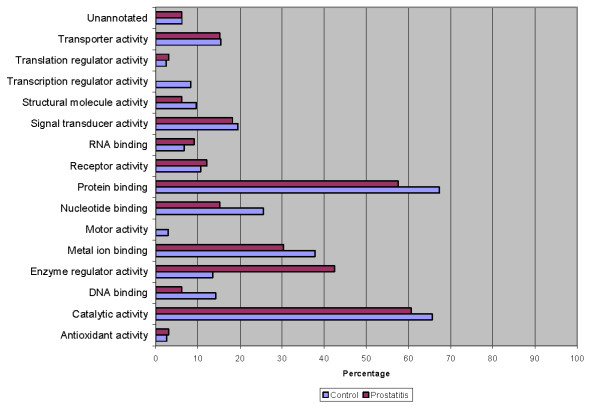
**Bar graph representing comparison of candidate proteins upregulated in prostatitis vs proteins found in normal healthy seminal plasma in terms of molecular functions**.

**Figure 7:**
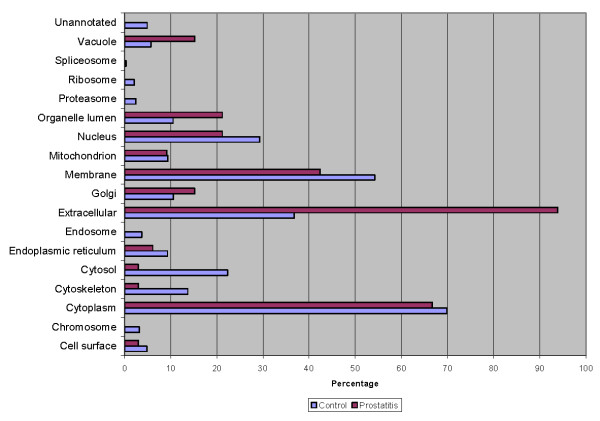
**Bar graph representing comparison of candidate proteins upregulated in prostatitis vs proteins found in normal healthy seminal plasma in terms of cellular components**.

**Figure 8:**
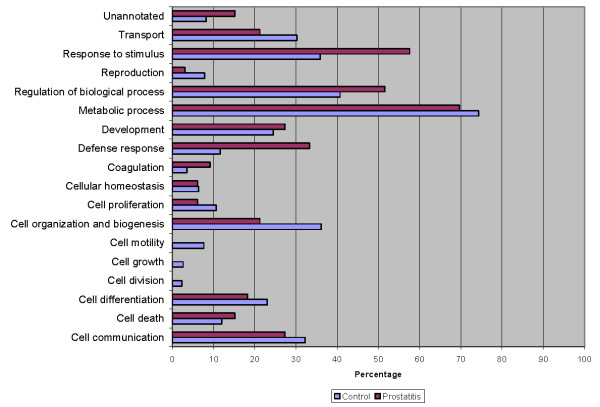
**Bar graph representing comparison of candidate proteins upregulated in prostatitis vs proteins found in normal healthy seminal plasma in terms of biological processes**.

**Figure 9:**
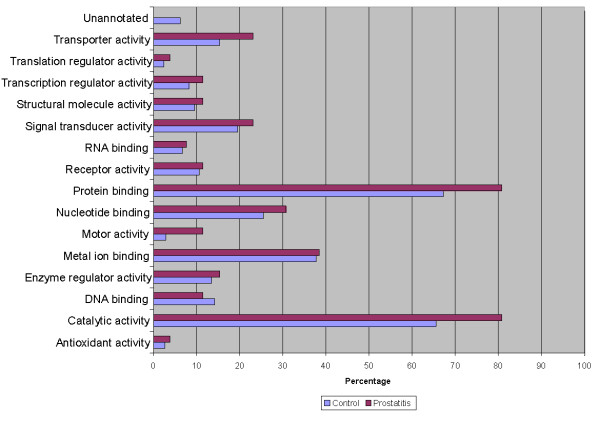
**Bar graph representing comparison of candidate proteins downregulated in prostatitis vs proteins found in normal healthy seminal plasma in terms of molecular functions**.

**Figure 10:**
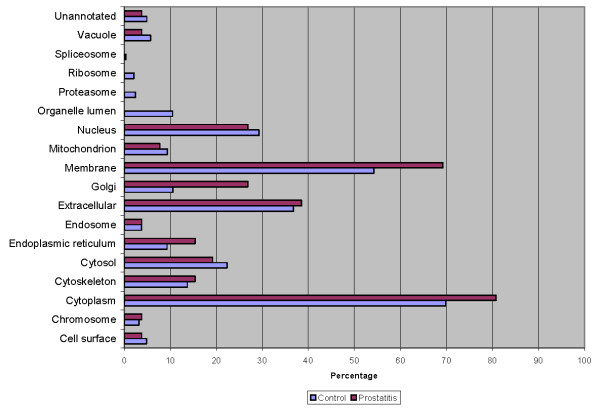
**Bar graph representing comparison of candidate proteins downregulated in prostatitis vs proteins found in normal healthy seminal plasma in terms of cellular components**.

**Figure 11:**
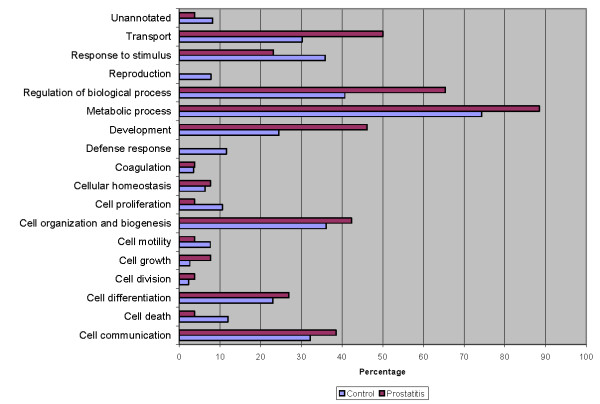
**Bar graph representing comparison of candidate proteins downregulated in prostatitis vs proteins found in normal healthy seminal plasma in terms of biological processes**.

To better delineate the differences between the normal seminal plasma proteome and those of prostatitis patients, a comparison of GO annotations was performed using ProteinCenter in which the seminal plasma proteome of healthy fertile controls [21] was compared to the protein candidates found to be upregulated (Figure 6, Figure 7, Figure 8) and downregulated (Figure 9, Figure 10, Figure 11) in prostatitis. In comparing the upregulated prostatitis candidates to the normal seminal plasma proteome, the most striking differences were a 3-fold increase in the percentage of proteins involved in enzyme regulatory activity, a 2.5-fold increase in the percentage of extracellular proteins, and a 3-fold increase in the percentage of proteins involved in the defensive response. A comparison of the candidates downregulated in prostatitis relative to the normal seminal plasma proteome showed a moderate (10-15%) increase in the percentage of proteins involved in catalytic activity and protein binding, a moderate (10-15%) increase in the percentage of proteins found in the cytoplasm, Golgi apparatus, and membrane, and a significant (20-25%) increase in the percentage of proteins involved in development, regulation of biological processes, and transport.

According to Ingenuity Pathway Analysis, the molecular and cellular functions associated with the largest number of candidate proteins are small molecule biochemistry, molecular transport, lipid metabolism, protein degradation, and protein synthesis. The diseases and disorders associated with the largest number of candidate proteins are neurological disease, cancer, endocrine disorders, metabolic disease, and reproductive system disease. The top canonical pathways most strongly represented by our upregulated candidates are acute phase response signaling, the complement system, and the coagulation system.

### Verification of Methodology

**Figure 12:**
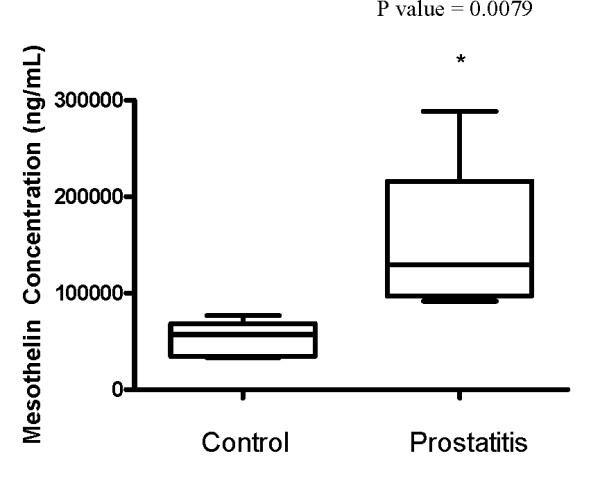
**Box plot representing ELISA candidate verification of mesothelin isoform 2**. * denotes significance (p < 0.05) as determined by two-tailed Mann Whitney test.

**Figure 13:**
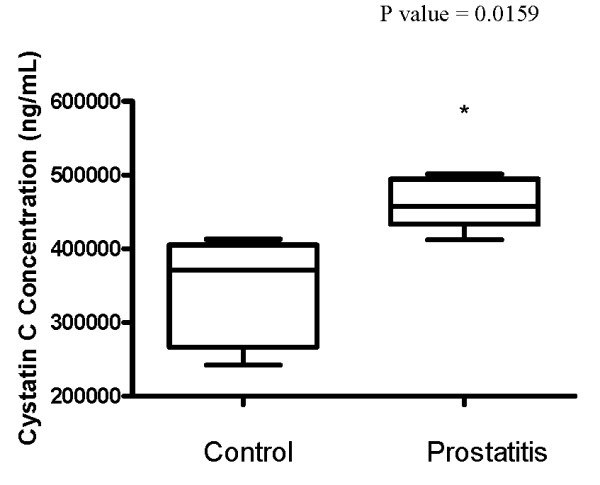
**Box plot representing ELISA candidate verification of cystatin C**. * denotes significance (p < 0.05) as determined by two-tailed Mann Whitney test.

Two candidate protein biomarkers were arbitrarily chosen based on fold differences (Table 1), putative roles in the pathogenesis of prostatitis, and the availability of assay reagents for verification using the gold standard in protein quantification, the enzyme-linked immunosorbent assay (ELISA). The proteins selected were mesothelin isoform 2 and cystatin C, both of which were upregulated in prostatitis samples compared to controls. Seminal plasma from each patient who contributed a sample to the mass spectrometric analysis was analyzed individually for mesothelin isoform 2 (Figure 12) and cystatin C concentrations (Figure 13). For mesothelin isoform 2, every sample from the Prostatitis group had a higher concentration than every sample from the Control group, and a mean concentration nearly 3 times greater in the Prostatitis group as compared to the Control group. Statistical analysis using a two-tailed Mann Whitney test found this difference to be significant (p = 0.0079). Similarly, Cystatin C was significantly increased in concentration in the seminal plasma of patients with prostatitis (p = 0.0159), although the difference was not as drastic. These results support our data obtained through mass spectrometric analysis, and suggest that the other candidates identified are likely upregulated or downregulated in prostatitis as compared to healthy fertile males. The discrepancy in fold change may be a consequence of the semi-quantitative nature of spectral counting with mass spectrometry, and reflects the need for future validation of other candidate biomarkers, using multiple reaction monitoring (MRM) or ELISA.

### Proteins Increased in Prostatitis

Of the 33 proteins increased in prostatitis, 26 were determined to be expressed in the prostate gland, according to at least one of three databases searched (UniGene, BioGPS, and the Human Protein Atlas). The 7 proteins that were not expressed in the prostate according to these databases included cystatin-S, cystatin-SA and lipocalin-1. However, cystatin-S gene expression in the prostate gland, as well as in other tissues of the male genitourinary system, has been reported [26]. As well, lipocalin-1, the major protein found in human tear fluid, has similarly been shown to be expressed in the prostate [27]. Thus, out of 33 candidate proteins from seminal plasma found to be increased in prostatitis, 28 are known to be produced in the prostate gland.

Protease inhibitors form a large proportion of the proteins found to be increased in the seminal fluid of prostatitis patients. The majority of these are members of the cystatin superfamily of secreted cysteine protease inhibitors, specifically the type 2 cystatin family. The type 2 cystatins found to be upregulated in prostatitis patients in our study are cystatin S, cystatin C, cystatin M, and cystatin SA. Other type 2 cystatins were previously found to be involved in processes such as spermatogenesis in the male reproductive tract, but those cystatins were not identified in our study [28]. While previous work on type 2 cystatins classifies their function as anti-inflammatory, and indicates that they are downregulated in inflammatory responses, conflicting evidence suggests that their regulation may not be so simple. Cystatin-C, a cysteine protease inhibitor, has been shown to be upregulated in Sjogren's syndrome, which is a paradigm of autoimmunity and inflammation [29]. Studies on inflammatory skin disorders also found increased levels of cystatin M/E in the spinous cell layers of inflammatory lesions, which, they speculated, might help control increased levels of cysteine proteases during inflammation and infection [30]. Similarly, production of cystatin-SA is enhanced by nuclear factor κ-light-chain enhancer of activated B cells (NFκB), an intracellular signaling molecule activated by pro-inflammatory cytokines [31]. Cystatin-SA, in turn, increases interleukin-6 production, which is a pleiotropic cytokine with many pro-inflammatory functions. One potential explanation for the upregulation of anti-inflammatory type 2 cystatin proteins in prostatitis is the body's attempt to regulate the inflammatory response and prevent excessive tissue destruction, similar to the case of activation of hemostatic pathways, which activate fibrinolytic proteins to prevent excessive clotting. Notably, none of the type 2 cystatins has previously been investigated as a biomarker of prostatitis, suggesting that they represent a promising target for future investigations.

Other protease inhibitors found to be increased in the seminal plasma of prostatitis patients in our study include SERPINA1 (alpha-1-antitrypsin), SERPINA5, SERPING1, SERPINF1, Lipocalin-1, and TIMP3. As with the type 2 cystatins, the literature generally characterizes these proteins as anti-inflammatory; however, this does not preclude their upregulation during inflammation. Lipocalin-1, for example, is another cysteine protease inhibitor that has been documented to be overexpressed during periods of stress, inflammation, or infection [32]. With the exception of SERPINA1, which has been found in histopathological studies of granulomatous prostatitis [33], none of these protease inhibitors have been studied in the context of prostatitis.

Other candidate biomarker proteins found in our experiments have functions more consistent with those expected to be upregulated during inflammation. Olfactomedin-4, for example, has been shown to be upregulated in crypt epithelial cells during inflammatory bowel disease [34], and is also induced by NFκB in myeloid precursor cells, suggesting that it forms part of a pro-inflammatory signaling network [35]. Alpha-2-macroglobulin and fibrinogen beta chain are acute phase proteins, which are known to be strongly upregulated during inflammation and other stresses [36]. Complement component C3 is a central player in the complement system, which is one of the major enzyme cascades activated during infection and inflammation [37]. Sphingomyelin phosphodiesterase isoform 1 has been described as strongly pro-inflammatory [38]. Mesothelin isoform 2, while not associated with inflammation in the literature, has been proposed as a biomarker to distinguish pancreatitis from pancreatic cancer [39]. Such a protein would be invaluable in differentiating other inflammatory and neoplastic processes (i.e. prostatitis and prostate cancer).

Of our 33 candidate biomarkers that were increased in prostatitis, only 5 have previously been studied for prostatitis diagnosis. Complement component C3 levels in seminal plasma were found to help distinguish chronic prostatitis and leukocytospermia from controls [12]. Urinary alpha-2-macroglobulin and albumin were investigated for possible utility in diagnosing acute prostatitis, and the ratio of alpha-2-macroglobulin:albumin was found to be a highly sensitive diagnostic test for acute prostatitis [40]. Furthermore, including other urinary parameters in a stepwise multinomial logistic regression analysis provides optimal differentiation between acute pyelonephritis and acute prostatitis [40]. Both SERPINA1 [33] and fibrinogen beta chain [41] have been found to be increased in prostatitis tissue obtained through biopsy, but the invasiveness of such a procedure is precisely what we are trying to avoid, by searching for a seminal plasma diagnostic biomarker. All of these previously investigated proteins are all quintessential pro-inflammatory proteins, which reflect our current understanding of prostatitis as an inflammatory disease of the prostate. By using a mass spectrometry approach to identify large numbers of proteins of diverse function, we can find proteins whose functions may be unrelated to inflammation, or are as yet unknown, but which still may function as biomarkers of prostatitis.

Comparing the candidates upregulated in prostatitis to the overall seminal plasma proteome in healthy fertile controls, reveals a striking 3-fold increase in the percentage of proteins annotated as possessing enzyme regulator activity, which is consistent with the number of candidates that regulate the inflammatory response. In terms of subcellular localization, the upregulated prostatitis candidates show an almost 3-fold increase in percentage of extracellular proteins, with a concomitant decrease in the number of cytoskeletal, cytosolic, and membrane proteins. Notably, while the percentage of membrane proteins in prostatitis patients' seminal plasma is decreased, compared to normal seminal plasma, it is still over 40% of the prostatitis candidates, which may reflect the cellular destruction in prostatitis and the release of membrane proteins into the seminal plasma. The biological processes of the seminal plasma proteomes from prostatitis vs control patients show a 3-fold increase in proteins involved in the defense response and coagulation, which supports our understanding of prostatitis as an inflammatory process. A moderate decrease (15%) in the percentage of proteins involved in cell organization and biogenesis is also noted, which may reflect the changing priorities of cells during an inflammatory response as compared to normal.

### Proteins Decreased in Prostatitis

A prostatitis biomarker could also be a protein that is decreased in the seminal plasma of prostatitis patients as compared to controls. Of our 59 candidate biomarkers, 26 were decreased in the seminal plasma of men with prostatitis. 25 of these were expressed in the prostate gland, according to at least one of three databases searched (UniGene, BioGPS, and the Human Protein Atlas). The one remaining protein is CACNA2D1, a voltage-dependent calcium channel subunit, which is not expressed in the prostate but is expressed in the testes (Unigene).

Candidate proteins which bear mention include SOD3 extracellular superoxide dismutase, which has been shown to be highly anti-inflammatory, inhibiting inflammatory cell migration by preventing fragmentation of hyaluronic acid [42]. Thus, the downregulation of SOD3 is consistent with prostatitis being a disease of inflammation. Moreover, previous studies have demonstrated that SOD3 levels in the seminal plasma are constant between fertile controls and vasectomized men, indicating that it originates proximal to the site of ligation of the vas deferens, likely the prostate [43]. Taken together, this suggests that SOD3 has potential in the diagnosis and monitoring of inflammatory diseases of the prostate.

Several other downregulated candidate proteins have been previously described as possessing pro-inflammatory properties. The most plausible explanation for this observation is the complex interplay between pro- and anti-inflammatory factors, resulting in a response that is carefully regulated and controlled. It is possible that our downregulated pro-inflammatory proteins are responding to regulatory signals, ensuring that the inflammation does not spiral out of control. Such pro-inflammatory proteins found in our study include Mucin-5b, which has been shown to be upregulated in inflamed nasosinus mucosa [44] and in chronic rhinosinusitis [45]. As well, Filamin-B, which functions to recruit inflammatory cells to the site of inflammation, was found to be decreased in expression [46].

Other noteworthy proteins include NOV and L-xylulose reductase, both of which are decreased in prostatitis, but which previous investigations have found to be increased in prostate cancer [47,48]. Both of these proteins could potentially be invaluable in distinguishing prostate cancer from prostatitis, which is currently a major confounder in the diagnosis of cancer.

Comparing the GO annotations of candidates downregulated in prostatitis to the normal seminal plasma proteome produced less striking results than the upregulated prostatitis candidates. This may reflect the fact that cells downregulate proteins of varying functions and localization during an inflammatory response, while upregulating proteins of a single function (i.e. defense). Compared to the normal seminal plasma proteome, the downregulated prostatitis candidates had moderate increases in the percentage of proteins involved in catalytic activity and protein binding. Downregulated prostatitis candidates also had increased percentages of proteins involved in regulation of biological processes, suggesting that while some regulatory proteins increase during prostatitis, others decrease. Notably, none of the downregulated prostatitis candidates were involved in the defense response, suggesting that prostatitis causes defensive proteins to be upregulated, not downregulated.

## Conclusion

We identified 59 proteins whose significantly differential expression in the seminal plasma of prostatitis patients suggests that they may have potential as biomarkers of prostatitis. Such proteins would have application in the diagnosis of prostatitis, as well as in monitoring response to treatment. Furthermore, improved methods of diagnosing prostatitis would better distinguish prostatitis from more serious causes of elevated PSA, such as prostate cancer, and reduce the need for unnecessary anxiety and invasive investigations. As well, diagnosing prostatitis could help better elucidate the cause of a patient's infertility. Many of our candidate biomarkers have previously been reported to be involved in inflammatory pathways, which correlate with our understanding of prostatitis as a disease of inflammation. A better understanding of the functions of these proteins may help to discover the pathogenesis of prostatitis, and improve our methods of treating it.

## Methods

Seminal plasma samples were collected, processed, and analyzed in accordance with the methods described elsewhere [21]. A brief summary of the methods for the Prostatitis and Control samples is given below.

### Sample Collection and Processing

Semen from men with prostatitis was collected after a minimum of 3 days of sexual abstinence. Our 5 patients all had confirmed prostatitis, as diagnosed by the presence of lower urinary tract symptoms (frequency, urgency, dysuria), pain in the pelvic region, and prostatic tenderness on digital rectal examination. Samples were allowed to liquefy, centrifuged, and the supernatant (seminal plasma) was stored for further analysis. Total protein concentration was measured using the Biuret assay. Five prostatitis seminal plasma samples were combined to make a "Prostatitis" pool with 3 mg total protein per experiment. Each sample contributed an equivalent amount of protein to the total pooled protein.

### Trypsin Digestion

Three "Prostatitis" replicate pools were denatured with urea, reduced with dithiothreitol and alkylated with iodoacetamide. Samples were then desalted, frozen and partially lypohilized. The samples were left to digest overnight using modified porcine trypsin (1:50, trypsin: protein concentration, Promega). Digestion was stopped the following morning by acidifying the solution with formic acid.

### Strong-Cation Exchange Liquid Chromatography

Samples were fractionated using an Agilent 1100 High Performance Liquid Chromatography (HPLC) system equipped with a strong-cation exchange (SCX) PolySULFOETHYL A column (The Nest Group, Inc.). Sixty fractions were collected. Fractions 31 to 49 were stored individually, while fractions 26-30 and 50-54 were pooled together (total 21 fractions).

### Mass Spectrometry

Twenty-one SCX fractions from each of the three Prostatitis replicates were further fractionated using nano-flow reverse-phase chromatography on a Proxeon EASY-nLC system, coupled online to a linear trap quadrupole (LTQ)-Orbitrap XL (Thermo Fisher Scientific, San Jose, California). For more details of the method, please see Batruch et al. [21]. The Orbitrap was used to acquire a full MS1 scan followed by six MS2 scans in the linear-ion trap (LTQ). Only charge states of 2+ and 3+ were subjected to fragmentation.

### Data analysis

X!Calibur RAW files were uploaded into Mascot Daemon (Matrix Science, London, UK, v.2.2) to create Mascot Generic Files (MGF). These were then searched against a concatenated forward and reverse IPI.Human v.3.54 database with Mascot (v.2.2) and X!Tandem (Global Proteome Machine Manager, version 2006.06.01) with a parent tolerance of 7 ppm and fragment tolerance of 0.4 Da. The following modifications were used in searches: one missed cleavage allowed, fixed carbamidomethylation of cysteines and the following variable modifications: oxidation of methionines, deamidation of asparagines and glutamines, cyclization of N-terminal glutamic and aspartic acids (pyroGlu-Asp), and protein N-terminal acetylation. Search result files from Mascot and X!Tandem were uploaded into Scaffold (Proteome Software, Portland, Oregon, v. 2.0) and filtered using peptide thresholds to achieve a false positive rate (FPR) of ~ 1.0-1.5%. FPR was calculated as follows: 2xFP/(FP+TP) where FP is the number of proteins matching the reverse database and TP is the number proteins matching the forward database.

### Verification of Methodology

In order to confirm the semi-quantitative data obtained from mass spectrometric analysis of the seminal plasma samples, 2 candidate proteins were arbitrarily selected for verification by enzyme-linked immunosorbent assay. The concentrations of cystatin-C and mesothelin isoform 2 were measured separately using specific sandwich immunoassays developed with DuoSet ELISA reagents (R&D Systems Inc., MN, USA). The cystatin-C assay employed two monoclonal antibodies, one for coating (Part 842942) and one for detection (Part 842943). Similarly, the mesothelin isoform 2 assay used two monoclonal antibodies, one for coating (Part 843359) and one for detection (Part 843360). All four antibodies were raised in mice. Both assays were conducted in a two-site sequential immunometric format with time-resolved fluorescence detection. Each assay has a detection limit of 0.1 ug/L and a dynamic range up to 10 ug/L. For each assay, within the measurement range, precision was < 15%. All standards and samples for both assays were measured in duplicate.

Prior to analysis, samples analyzed for cystatin-C were diluted 10,000 times in a dilution buffer consisting of 1% bovine serum albumin (BSA) in phosphate-buffered saline (PBS) at pH = 7.2. Samples analyzed for mesothelin isoform 2 were diluted 100 times in the same buffer.

Each well on the cystatin-C plate was incubated overnight at room temperature with 250 ng/100 uL monoclonal capture antibody in coating buffer (PBS, pH = 7.2). Each well on the mesothelin isoform 2 plate was incubated overnight at room temperature with 400 ng/100 uL monoclonal capture antibody in coating buffer (PBS, pH = 7.2). The next day each plate was washed 3 times with washing buffer (PBS with 0.05% Tween 20 at pH = 7.4). Dilution buffer (1% BSA in PBS at pH = 7.2) was then added to each well to block the plate for 1 hour. Each plate was then washed 6 times with washing buffer and then 100 uL of diluted samples or standards were added to each plate. Both plates were then incubated at room temperature for 90 minutes with gentle shaking. Each plate was then washed 6 times with washing buffer. To each well on the cystatin-C plate, 25 ng/100 uL of biotinylated detection antibody in dilution buffer was added. To each well on the mesothelin isoform 2 plate, 100 ng/100 uL of biotinylated monoclonal anti-human mesothelin isoform 2 detection antibody in dilution buffer was added. Both plates were then incubated for 1 hour at room temperature with gentle shaking, then washed 6 times with washing buffer. Five ng/well of alkaline phosphatase-conjugated streptavidin solution (Jackson ImmunoResearch) in sample buffer was added to each well, and allowed to incubate at room temperature for 15 minutes with gentle shaking. Each plate was then washed 6 times with washing buffer. To each well on both plates 100 μL of substrate buffer [0.1 mol/L Tris buffer (pH 9.1) containing 0.25 mmol/L diflunisal phosphate (DFP), 0.1 mol/L NaCl, and 1 mmol/L MgCl_2_] was added. Both plates were then incubated at room temperature for 10 minutes with gentle shaking. To each well on both plates 100 uL of developing solution (1 mol/L Tris base, 0.15 mol/L NaOH, 2 mmol/L TbCl_3_, 3 mmol/L EDTA) was added and allowed to incubate for 1 minute at room temperature with gentle shaking prior to measurement. Protein concentration was quantified by measuring fluorescence in each well with an Envision time-resolved fluorometer (Perkin Elmer) as previously described [49].

## Abbreviations

ELISA: Enzyme-linked immunosorbent assay; PSA: Prostate-specific antigen; HPLC: High performance liquid chromatography; SCX: Strong cation exchange; LTQ: Linear trap quadrupole/linear ion trap; MGF: Mascot generic file; FPR: False positive rate; FP: False positive; TP: True positive; MS: Mass spectrometry; BSA: Bovine serum albumin; PBS: Phosphate buffered saline; DFP: Diflunisal phosphate; CV: Coefficient of variance; GO: Gene ontology; MRM: Multiple reaction monitoring.

## Competing interests

The authors declare that they have no competing interests.

## Authors' contributions

DK participated in sample preparation, mass spectrometry, data analysis, verification of methodology, and drafted the manuscript. IL participated in sample preparation, mass\spectrometry, data analysis, and statistical analysis. IB designed the methodology, and led the sample preparation, mass spectrometry, and data analysis, as well as contributing substantially to the manuscript. CS ran the mass spectrometer and assisted in data analysis. IK, KL, and EG conceived of the study and participated in patient recruitment. ED and KJ ran the lab in which the study occurred, conceived of the study, designed the study, recruited patients, and coordinated the entire project, as well as being substantially involved in the editing of the manuscript. All authors read and approved the final manuscript.

## Supplementary Material

Additional File 1 **Proteins identified in triplicate analysis of Prostatitis seminal plasma samples**. This file contains all proteins identified in Prostatitis group seminal plasma along with the number of spectra, number of unique peptides and protein sequence coverage in each replicate. Click here for file

Additional File 2 **Proteins identified in triplicate analysis of Control and Prostatitis seminal plasma samples**. This file contains all proteins identified in Control and Prostatitis group seminal plasma along with number of spectra in each replicate. Click here for file

Additional File 3 **Instructions for retrieving Additional File **3. This file contains instructions on how to download three Spectrum report files (1of3, 2of3 and 3of3) from Tranche (http://www.ProteomeCommons.org). The three Spectrum report files contain information about all peptides that were identified in Prostatitis group seminal plasma, such as: strong-cation exchange fraction in which the peptide was identified, Mascot ion and X!Tandem peptide scores, peptide modifications, observed m/z. Click here for file
