# Supplementary material for: Characterization of the seminal plasma proteome in men with prostatitis by mass spectrometry
Source: Clin Proteomics. 2012 Feb 6;9(1):2. doi: 10.1186/1559-0275-9-2 (PMC3305567; doi:10.1186/1559-0275-9-2)
Supplement: Additional File 3 — Instructions for retrieving Additional File 3. This file contains instructions on how to download three Spectrum report files (1of3, 2of3 and 3of3) from Tranche (http://www.ProteomeCommons.org). The three Spectrum report files contain information about all peptides that were identified in Prostatitis group seminal plasma, such as: strong-cation exchange fraction in which the peptide was identified, Mascot ion and X!Tandem peptide scores, peptide modifications, observed m/z. [file 1559-0275-9-2-S3.DOC]

**Additional File 3 - instructions**

1) Go to the following websites (each website corresponds to a data file):

**Part 1 of 3:** <https://proteomecommons.org/tranche/data->

downloader.jsp?fileName=ZHUIz%2BLBU3tDllcBXq0fOcfIjNkLwJQHorwem1aTQgnIxlgh%2Br68xTk%2FtwguZZb02xLntiL%2FExIkd8jlIeorU4ePHYsAAAAAAAAC0A%3D%3D

**Part 2 of 3**: <https://proteomecommons.org/tranche/data->

downloader.jsp?fileName=pvg3sXlXYw7xwG%2FGVAg%2FUAm033UnGZGf2I7Ll44C%2BKZWYIytNS3u%2BJAui3jdQY70jweURTU7uAJX9TbYdefGyzSsmloAAAAAAAAC0A%3D%3D

**Part 3 of 3**: <https://proteomecommons.org/tranche/data->

downloader.jsp?fileName=4sPrv2Fe50RQMRWuVd2ACLjycum%2BFkzibA5HFE6cp%2BifMLsewmXesq8KPmgvOYMYSdcNGmk4E%2FAnzzHn1pe3TWg%2Fl7AAAAAAAAAC0A%3D%3D
